# Evaluation of Validity and Reliability of a German General and Sports Nutrition Knowledge Questionnaire for Athletes and Coaches (GSNKQ-AC)

**DOI:** 10.3390/nu15224844

**Published:** 2023-11-20

**Authors:** Helen Bauhaus, Pia Malin Jensen, Hans Braun, Mario Thevis

**Affiliations:** 1 Institute of Biochemistry, German Sport University Cologne, 50933 Cologne, Germany; pia@piajensen.de (P.M.J.); thevis@dshs-koeln.de (M.T.); 2 German Research Centre of Elite Sports, German Sport University Cologne, 50933 Cologne, Germany; h.braun@dshs-koeln.de; 3 Manfred Donike Institute for Doping Analysis, German Sport University Cologne, 50933 Cologne, Germany; 4 Centre for Preventive Doping Research, German Sport University Cologne, 50933 Cologne, Germany

**Keywords:** validation, questionnaire, sports nutritional knowledge, athletes and coaches, diet, knowledge assessment

## Abstract

Despite the availability of nutritional recommendations, studies have reported inadequate nutrition in athletes. The existing literature highlights the importance of the nutritional knowledge of both athletes and coaches in influencing athletes’ food choices and behavior, as well as its direct and indirect impact on athletes’ performance and health. To adequately assess nutritional knowledge, monitoring via valid and reliable questionnaires is required. As no questionnaire tailored to German-speaking athletes and coaches exists, this study aimed at developing a new General and Sports Nutritional Knowledge Questionnaire for Athletes and Coaches (GSNKQ-AC). The development followed a literature-based, ten-step validation approach. The initial questionnaire (63 items) was revised and reduced to 29 items in the final version after conducting construct verification in the target group (*n* = 84 athletes and coaches), evaluating content validity by a panel of nutrition experts (*n* = 8), verifying face validity by think-out-loud interviews in the target group (*n* = 7), and conducting classical test theory for item reduction analysis (*n* = 53). For the final GSNKQ-AC, internal consistency, calculated as Cronbach’s alpha, was 0.87. Students with a focus on sports nutrition (*n* = 31) scored significantly higher than athletes and coaches (*n* = 53), revealing good construct validity (77% vs. 62%, *p* < 0.001). Test–retest reliability (*n* = 42, matched pairs) showed a Spearman’s correlation coefficient of r = 0.61 (*p* < 0.01). The brief GSNKQ-AC can be used for status quo or longitudinal assessment of nutritional knowledge among athletes and coaches to reveal gaps and ensure purposeful planning of educational interventions.

## 1. Introduction

Nutritional knowledge has been reported to influence food choices and behavior [1,2]. This knowledge can lead to meeting general food intake recommendations when food and nutrition literacy is higher [3,4,5]. At the same time, nutritional status, directly and indirectly, affects athletic performance [6,7]. Therefore, general nutritional recommendations for athletes aim to maintain their health, promote training adaptations, and support recovery [7]. Unlike existing theoretical recommendations and guidelines, the literature reports insufficient nutritional status among athletes [8,9,10]. Most data show lower carbohydrate (CHO) and energy intake, while protein (PROT) and fat intake tended to be higher than recommended. Consideration of fueling for and recovering from intensive training sessions and competition is crucial. Nutrient intake before (four hours) as well as during and after (two hours) the latter is crucial and part of an individualized nutritional strategy of an athlete. Internal data from different soccer youth academies [11] considering fluid, CHO, and protein intake before, during, and after training or matches show inadequate fluid and CHO intake at all timepoints and low PROT intake after training and competition compared to consensus-based recommendations [12]. From researchers’ and practitioners’ perspectives, operating competence in (sports) nutrition offers a chance to improve athletes’ knowledge and therefore optimize nutritional status [13]. It is hypothesized that improving nutritional knowledge through education would help athletes adhere to a sports-appropriate diet [14]. Several studies and reviews have reported an association between nutritional status and dietary intake [4,15,16]. In an athletic population, an association between knowledge and positive dietary attributes was found in five out of seven studies [16] and six out of nine studies [4]. Conversely, there is no association between poor nutritional knowledge and negative dietary attributes [16]. Compared with studies conducted in the general population, the prevalence of a positive association between nutritional knowledge and positive dietary attributes was higher in studies conducted in an athletic population: 71.4% of reviewed studies focusing on athletes showed a positive association compared to 63.6% of reviewed studies focusing on the general population [16]. In order to evolve the current state or measure the effectiveness of educational programs through pre- and post-evaluations, questionnaires can be used; they are easy tools that require low compliance, low cost, and little time. Nevertheless, criticized the application of partly or fully non-validated tools to investigate nutritional knowledge and status is criticized [4]. In addition to a lack of validation, further limitations of questionnaires are mentioned [17]. These include referring to outdated recommendations, showing a lack of comprehensiveness, and not being adapted to the cultural aspects of respondents. When searching for questionnaires, we found validated questionnaires in different languages, such as Portuguese, Finnish, English, and Spanish [18,19,20,21] and many non-validated questionnaires that are considered critical in terms of producing valid and reliable results [22]. To evaluate the nutritional knowledge of German athletes, it is necessary to apply a questionnaire in the German language. We found one questionnaire that addresses German athletes [23]. The questionnaire covers general nutritional recommendations and includes the topic of diet-related diseases, while sports nutrition recommendations and sports nutrition–related topics such as dietary supplements and fluid management are not considered, resulting in limited application for elite athletes. Sports nutritional knowledge is defined as “knowledge of concepts and processes related to nutrition for optimal athletic performance including knowledge of weight management; hydration and fueling strategies for before, during and after training/performance; supplementation and alcohol use” [24]. Therefore, the aim of this article is to present the development and robust validation process of a new *General and Sports Nutritional Knowledge Questionnaire for Athletes and Coaches* (GSNKQ-AC) in the German language, covering the following topics, each referring to public and sports nutritional recommendation guidelines: energy intake and weight management, macronutrients, and specific knowledge, including questions about micronutrients, hydration management, and dietary supplements. Since some studies have shown an association between coaches’ and athletes’ knowledge [25,26,27], the questionnaire was validated in both groups. Exercise Science students with special education in sports nutrition represented the focus group of the validation process.

## 2. Materials and Methods

**Figure 1:**
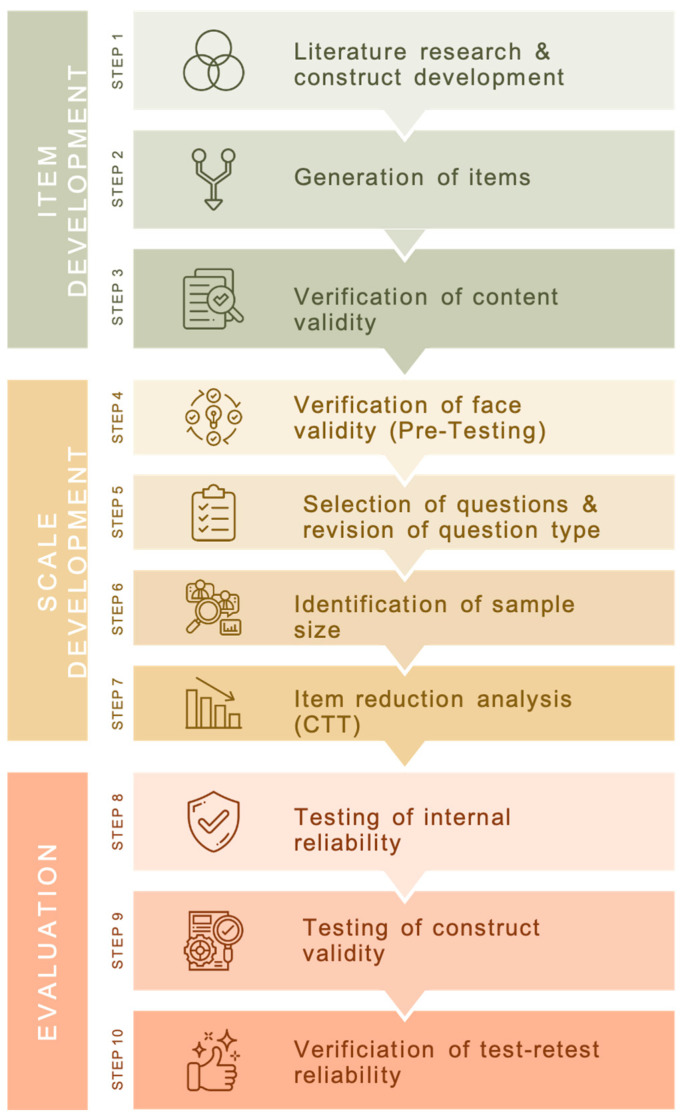
Flowchart showing the ten-step validation model of the General and Sports Nutritional Knowledge Questionnaire for Athletes and Coaches.

The development and validation of the questionnaire were mainly based on selected studies [19,24,28,29,30]. Consistent development and validation steps were identified. These led to a three-phase model with a ten-step validation process for the new questionnaire. An overview of the validation model is shown in Figure 1.

### 2.1. The Ten-Step Validation Process

(1) Step one, literature research, was conducted to identify relevant fields that need to be covered by the questionnaire. (2) In the second step, items were generated according to the topic. In order to record inter-individual differences with regard to a characteristic, it is necessary to generate items that are representative of the related knowledge characteristic [28,31,32]. For the questionnaire, items were generated in a deductive manner using a fixed-answer format and single-choice answer option. (3) Adequacy of items was evaluated by content validity. Content validity assesses relevance, clarity, correctness, and appropriateness among sports nutrition experts working as researchers and/or practitioners (*n* = 8) [28,33]. (4) Face validity was assessed using think-out-loud interviews conducted with a small sample (*n* = 7) representing the target group [24,34]. (5) The selection and revision of questions and question types were conducted based on the results of steps three and four [28]. (6) A priori determination of the sample size was the basis for conducting tests seven to ten. (7) Item reduction analysis was conducted using classical test theory (CTT) [35]. (8) Internal reliability was assessed using Cronbach’s alpha to evaluate the internal consistency of the scale items. A Cronbach’s alpha coefficient of 0.70 is considered to be an acceptable threshold for reliability; however, values between 0.80 and 0.95 are preferred for psychometric quality of the scales [28]. (9) Construct validity was verified using differentiation by group as an indicator, which compares the scores of participants with specific knowledge (focus group) to those without specific knowledge (target group). For test–retest reliability (10), the survey was sent out to the participants a second time. Total and sub-section test scores of the first and second survey were correlated in a matched-pair manner. The two surveys are recommended to be separated by at least two weeks [29].

### 2.2. Resources and Recruitment of Experts and Subjects

The questionnaire was distributed online via UNIPARK (Tivian IX GmbH, Cologne, Germany). Sports nutrition experts from the authors’ networks were contacted and asked to provide feedback as required for step three. In total, eight experts agreed to comment and discuss the questionnaire. For step four (think-out-loud interviews), seven athletes competing at least on a regional level and having not yet received any nutritional consultation and therefore having no considerable background in nutrition were recruited from different sports clubs in Cologne, facilitating in-person meetings. For steps eight to ten, participants were recruited for either the focus or target group. The focus group included students with special knowledge in sports nutrition recruited from the German Sport University Cologne (survey 1: *n* = 31; survey 2: *n* = 23). The target group included athletes (survey 1: *n* = 34; survey 2: *n* = 16) and coaches (survey 1: *n* = 19; survey 2: *n* = 12) from various sports. Recruitment of the target group was done by contacting coaches directly, e.g., by issuing an inquiry for forwarding the questionnaire to their teams/athletes. Prior to the survey, participants were informed of the purpose of the study, and they declared consent. Anonymous data were collected between March and July 2021.

### 2.3. Statistical Analysis

Data analysis was conducted using R version 4.1.3 (The R Foundation for Statistical Computing) and Excel 2019 (Microsoft Corp., Redmond, WA, USA). Data are presented as means ± standard deviation. Before performing parametric or non-parametric tests, the normality of residuals was tested using the Shapiro–Wilk test as well as QQ-plots and histograms. As normality of the data was not given, the Mann–Whitney U test was performed for assessing construct validity. Spearman’s correlation coefficient and the Wilcoxon test was performed for reliability testing.

## 3. Results

### 3.1. Questionnaire Development, Generation of Itemsm and Pilot Testing

The initial pilot version of the questionnaire consisted of 63 items (steps 1 and 2). The results of the pilot survey showed insufficient internal reliability and a lack of difficulty. This was indicated if more than 90% of participants answered an item correctly. Comprehensive revision included changes in question format, deletion of 13 questions, and alignment of eight questions (steps 3 and 4). The revised questionnaire included 50 items in total, with five sub-categories: energy and weight management (*n* = 12), macronutrients (*n* = 26), micronutrients (*n* = 3), fluid balance (*n* = 5), and dietary supplements (*n* = 4). The 50-item version was evaluated according to the steps described in the methods.

### 3.2. Content Validity

Content validity of the revised version (step 3) was re-evaluated by the same eight nutrition experts who had already evaluated the pilot version of the questionnaire. Items that seemed imprecise, irrelevant, or redundant were either improved (e.g., rephrasing of questions/answers, changing order) or removed (*n* = 11). Face validity (step 4), which was conducted as a think-out-loud interview of subjects representing the target group (*n* = 7), led to rephrasing of questions and answers to improve the comprehensiveness of the questionnaire.

### 3.3. Sample Size Determination

The sample size (step 6) for item reduction analysis was chosen to be equal to the number of questionnaire items plus one [29]. In the case of the present questionnaire, sample size for item reduction analysis should be 51. For parametric and non-parametric testing, a priori power analysis was performed using G*Power version 3.1.9.6 [36] with alpha = 0.05, power = 0.80, and Cohen’s d = 0.80 [37]. For a two-tailed independent *t*-test, power analysis yielded a sample size of 26 participants each for target and focus groups. For a two-tailed dependent *t*-test, the power analysis yielded a sample size of 15. The characteristics of participants are specified in Table 1.

### 3.4. Item Reduction Analysis

Item reduction analysis considers an item’s difficulty and selectivity. Item difficulty was evaluated on the basis of the percentage of correct answers of 53 athletes and coaches. Questions that were answered correctly by less than 10% and more than 90% of all participants were considered too difficult or too easy, respectively. Item selectivity required at least r = 0.2. Exceptions were made when the expert panel considered the item important and type of phrasing the question appropriate. The item difficulty and selectivity results are shown in Table 2. The results of difficulty and selectivity were again discussed with the expert panel.

### 3.5. Internal Reliability and Construct Validity

Internal reliability, calculated as Cronbach’s alpha, for the total questionnaire and sub-sections ranged from 0.44 to 0.82 (Table 3). As a result of calculating internal reliability, the initial five sub-sections were reduced to three sub-sections: (1) Energy and Weight management, (2) Macronutrients, and (3) Specific Knowledge, which included questions about micronutrients, supplements, and fluid intake/management. Evaluation of construct validity revealed a significant difference between focus (*n* = 31) and target groups (*n* = 53) (Table 4).

### 3.6. Test–Retest Reliability

For verifying test–retest reliability, the questionnaire was sent to the same participants two weeks after they received the questionnaire the first time. The survey was accessible for four weeks. Answers were received between the second and fourth week, so that the two tests were separated by four to six weeks. After matching the datasets of the first and second surveys of each subject, 42 complete datasets were available for reliability testing. Spearman’s correlation of nutritional knowledge score was significant for the total questionnaire and for each sub-category, with a high to moderate correlation (Table 3).

## 4. Discussion

### 4.1. Validation Model and Results

Nutrition and food literacy should be part of an athlete’s interdisciplinary education to develop responsibility [38]. A valid and reliable tool is necessary to assess the efficacy of an educational program [17]. A validated questionnaire is low cost and requires little effort from an athlete’s perspective (10 to 15 min) and minor statistical knowledge from a researcher’s or practitioner’s perspective, as it produces meaningful data by calculating a nutritional knowledge score. The score allows for intra- or inter-comparison of an individual athlete or group/team. For a German athletic cohort, only one validated questionnaire is available [23]. This questionnaire includes questions regarding general recommendations and disease-related questions. In line with international literature and validated questionnaires from other countries such as Brazil, Finland, Australia, and Spain [18,19,20,21], the recently developed 29-item questionnaire covers general nutrition– and sports nutrition–related topics: (1) energy and weight management, (2) macronutrients, and (3) specific knowledge (micronutrients, hydration management, and dietary supplements). Questions are asked in a close-ended manner. Open-ended questions cannot be consistently coded and therefore are not able to produce reliable data [24]. Questions and answers are based on the current literature and sports nutritional guidelines [6,7,12,39,40]. The herein presented validation process followed a ten-step validation protocol, which is the result of a comprehensive literature review [19,24,28,29,30] (Figure 1). Instead of face validity (step 4), which was conducted using a think-out-loud method following Buehler’s psychology of thought (1907), written responses in the first version can also be evaluated as well [29]. The current validation process implemented the think-out-loud method, as it allows instant feedback on items by following the cognitive processes of the respondent’s problem solving. At the same time, it requires more time, as the researcher needs to conduct an interview with each subject in the think-out-loud sample. The method has been used in various areas of pedagogical–psychological and didactic teaching–learning research and was therefore applied in this study despite the aspect of increased duration [30,34]. Verification of content validity (step 5) includes not only nutritionists but also psychologists in the expert panel [29]. Instead of psychologists, the current validation study conducted content validity tests with nutritionists who are working as researchers but also as practitioners. We consider practitioners to be very important for the verification of content validity, as they work with the target group on a daily basis. All of the eight experts have an academic background in sports nutrition. Three to ten experts are recommended for the size of the panel [33], which complies with the sample size of experts chosen in this study. While some literature recommends Rasch Analysis for item reduction analysis [30], the current validation process performed item reduction analysis by calculating classical test theory [28,29] (step 7). Classical test theory has been shown to produce stable results for sample sizes of 30–50; Rasch analysis seems to require a sample size larger than 100 [41,42]. Internal reliability (step 8) is rated good when Cronbach’s alpha is at least 0.8, which is given for the total questionnaire (Cronbach’s alpha = 0.82). Sub-sections did not achieve an acceptable Cronbach’s alpha (≥0.7), which is due to the small number of items [29,43]. The Spearman’s correlation coefficient for the total questionnaire is r = 0.61, ranging from r = 0.552 to r = 0.74 in the sub-sections. A correlation coefficient of at least 0.7 is recommended [28]. According to this threshold, only the third sub-section “Specific Knowledge” reaches a sufficient correlation coefficient, higher than 0.7 (r = 0.74). Other questionnaires show higher correlation coefficients for test–retest reliability with r = 0.92 testing ten days to two weeks apart from the initial test [24], r = 0.85 with a high variability between sub-sections (r = 0.49 up to r = 0.87) testing five weeks apart [19], and r = 0.895 ranging from r = 0.53 to r = 0.81 in sub-sections testing two to four weeks apart [21]. One of the aforementioned studies conducted their reliability test mainly in their focus group (53% vs. 45% in our retest sample) [19], who were expected to have higher knowledge and therefore might answer more similarly on two test occasions than participants without pre-existing knowledge. The time between the first and the second survey should not be long enough for participants to gather new knowledge on the questionnaire items but also not short enough for them to be able to remember their given responses in the first round. Generally, two weeks between two identical surveys is considered practical and was also intended to be applied in this study. Due to the response time of the participants, the actual time between the two surveys was four to six weeks, which could have influenced the results. Reliability testing is limited by the fact that motivated subjects could look up the answers to items, which affects correlation negatively [24]. In the case of our study, we cannot exclude that subjects were informing themselves during the prolonged time period between the two surveys, which could have led to a correlation coefficient < 0.7. Also, the lower correlation coefficient can be explained by the fact that some items in the questionnaire did not meet the difficulty index but were kept in the final questionnaire version, as the expert panel considered them important. All validation steps included revision of the questionnaire based on the results of the first test, followed by test–retest analysis for final validation. Revisions included the exclusion of items, change of items, change of responses, change of response scale, rechecking content validity, internal consistency, and construct validity.

### 4.2. How Could the Questionnaire Be Applied in the Future in Practice and Research?

Consistent use of the same valid and reliable tool is necessary to compare results of an individual, of groups, and between different studies [4]. Therefore, international comparisons are and will be difficult to assess, as each country should adopt questionnaires for their international food habits and culture [24,30]. However, this questionnaire is, with permission, like many others [18,19,21], based on a recently developed and validated questionnaire [44] and considered the most recent standard for sports nutritional guidelines. Hence, it appears fair to assume that this ensures the best possible international comparability. A first survey with the final version of the GSNKQ-AC of different German national soccer youth teams (*n* = 72 female players, *n* = 63 male players) showed a nutritional knowledge score of 60 ± 15% (females: 68 ± 11% vs. males: 51 ± 14%, *p* < 0.001) (Van der Felden & Bauhaus, unpublished data). These scores are in line with scores found internationally, with a nutritional knowledge score of 51% for Australian Football players [45], 43% in youth academy players [46], 46 ± 12% in female Gaelic football players [47], and 58 ± 19% in division I college athletes, with females scoring higher than males (67 ± 16% vs. 46 ± 15%, *p* < 0.001) [48]. The correlation between nutritional status and nutritional knowledge can be determined by correlating the overall score with overall nutritional intake. Sub-sections can also be correlated (e.g., knowledge score on the topic “macronutrients” and actual intake of PROT, CHO, and fat). As athletes make their food choices inter alia based on nutrient composition, ingredient lists, allergens, and food labels, food and nutrition literacy are required [49]. It should still be considered that nutritional status is not only dependent on nutritional knowledge but also on behavioral, socioeconomic, and motivational aspects [1,50,51], which are not assessed by this questionnaire. In addition, correlating nutritional status and knowledge is challenging because of the social desirability bias of self-reported food intake and unintentional misreporting [52,53]. Therefore, the correlation between the nutritional knowledge scores and nutritional status poses a risk of bias. As the literature shows a relationship between athletes’ and their coaches’ nutritional knowledge [14], and athletes commonly mention their coaches as one of their main sources of nutritional advice [54,55], the questionnaire was validated in both groups independently of their sport.

### 4.3. Limitations

The sample of athletes of the present validation is skewed towards track and field as well as wrestling athletes. The sample was intended to be more heterogenous. Therefore, the questionnaire was sent to individual athletes and intermittent-based sport teams such as hockey, handball, and badminton. After sending several reminders, the quantity and quality of responses was low in these sports. The aforementioned survey in the German national soccer youth teams confirmed this tendency towards low responsiveness. The survey link was sent out to ten national youth teams in total. Only 135 of circa 250 players fully answered the survey. In the national youth team’s survey, interest in and attitude towards sports nutrition were added as questions at the end of the questionnaire. Only 66% of this sample indicated that they were interested in sports nutrition, although 84% believe that sports nutrition has a crucial impact on performance (van der Felden & Bauhaus, unpublished data). In contrast, four players believe that nutrition has no impact at all. Similar data could not be found. Challenges in assessing nutritional knowledge data and relatively low interest in nutrition in team sports athletes, particularly in soccer players, are described elsewhere [21]. The relatively low interest in sports nutrition might explain the present validation sample despite the attempt to include various sports.

## 5. Conclusions

In conclusion, a validation model for nutritional knowledge questionnaires is presented and considered fit-for-purpose, as demonstrated by the development of a new General and Sports Nutritional Knowledge Questionnaire for Athletes and Coaches for German speaking participants. The questionnaire allows assessment of knowledge gaps concerning the following topics in athletes’ nutrition: energy balance, weight management, macronutrient recommendations on a daily basis and nutrient timing for athletes, micronutrients, hydration management and fluid intake, and dietary supplements. Each section is conceptualized in a way that knowledge about recommendations and knowledge about foods and their nutritional value is assessed. The questionnaire is applicable to status quo assessments and can further be used for longitudinal assessments (e.g., development of knowledge after a year) of natively German-speaking athletes and coaches. The validated survey form should be used as it is or with minimal modifications, as described by elsewhere [22]. Further questions can be added at the end.

## Figures and Tables

**Table 1:** Anthropometric data of participants of the first test (a) and second retest (b) survey. (S&C = strength & conditioning).

(a)		Total	Students	Athletes	Coaches
*n*		84	31	34	19
Gender	f	40	11	23	6
	m	44	20	11	13
Age	mean	27.5	25.5	23.8	37.6
	sd	10.9	2.29	11.8	11.8
Sports	Track &field	28	4	19	5
	Triathlon	6	3	1	2
	Swimming	7	2	2	3
	Cycling	3	3	0	0
	Gymnastics	2	2	0	0
	S&C	3	3	0	0
	Karate	2	0	0	2
	Wrestling	12	0	11	1
	Canoeing	1	0	0	1
	Soccer	3	3	0	0
	Handball	4	0	1	3
	Tennis	1	0	0	1
	Badminton	1	1	0	0
	Fitness	8	8	0	0
	NA	2	2	0	0
Level	Regional	39	24	14	1
	State	10	3	5	2
	National	19	1	11	7
	International	16	3	4	9
**(b)**		**Total**	**Students**	**Athletes**	**Coaches**
*n*		51	23	16	12
Gender	f	22	10	9	3
	m	29	13	7	9
Age	mean	29.5	25.3	29.1	37.9
	sd	9.9	2.4	10.1	13.5
Sports	Track & field	21	2	15	4
	Triathlon	4	2	1	1
	Swimming	2	1	0	1
	Cycling	3	3	0	0
	Gymnastics	2	2	0	0
	S&C	3	3	0	0
	Karate	1	0	0	1
	Wrestling	1	0	0	1
	Canoeing	1	0	0	1
	Soccer	2	2	0	0
	Handball	3	1	0	2
	Hockey	0	0	0	0
	Tennis	1	0	0	1
	Badminton	2	2	0	0
	Fitness	5	5	0	0
	NA	0	0	0	0
Level	Regional	25	18	7	0
	State	5	0	4	1
	National	10	1	4	5
	International	11	4	1	6

**Table 2:** Item difficulty and selectivity of the 50-item questionnaire that was initially validated. Bold items labelled with an asterisk were kept in the final version of the questionnaire after the validation process.

Item	Scale	Item Difficulty (%)	Item Selectivity (%)
q01	Energy and Weight Management	0.92	0.49
q02 *	**Energy and Weight Management**	**0.64**	**0.25**
q03	Energy and Weight Management	0.95	−0.02
q04	Energy and Weight Management	0.99	0.34
q05	Energy and Weight Management	0.98	0.51
q06 *	**Energy and Weight Management**	**0.77**	**0.22**
q07 *	**Energy and Weight Management**	**0.87**	**0.33**
q08 *	**Energy and Weight Management**	**0.68**	**0.2**
q09 *	**Energy and Weight Management**	**0.42**	**0.37**
q10 *	**Energy and Weight Management**	**0.52**	**0.17**
q11	Energy and Weight Management	0.93	0.31
q12	Energy and Weight Management	0.38	0
q13	Macronutrients	1	0.28
q14 *	**Macronutrients**	**0.27**	**0.47**
q15 *	**Macronutrients**	**0.68**	**0.56**
q16 *	**Macronutrients**	**0.9**	**−0.25**
q17	Macronutrients	0.65	0.26
q18 *	**Macronutrients**	**0.55**	**0.19**
q19 *	**Macronutrients**	**0.19**	**0.03**
q20	Macronutrients	0.42	0.45
q21 *	**Macronutrients**	**0.75**	**0.17**
q22 *	**Macronutrients**	**0.3**	**0.46**
q23 *	**Macronutrients**	**0.9**	**−0.06**
q24	Macronutrients	0.89	0.32
q25 *	**Macronutrients**	**0.67**	**0.19**
q26	Macronutrients	0.92	0.30
q27 *	**Macronutrients**	**0.57**	**0.06**
q28	Macronutrients	0.98	0.38
q29 *	**Macronutrients**	**0.8**	**−0.02**
q30	Macronutrients	0.69	0.31
q31 *	**Macronutrients**	**0.75**	**0.49**
q32 *	**Macronutrients**	**0.75**	**0.44**
q33 *	**Macronutrients**	**0.86**	**0.1**
q34	Macronutrients	0.93	0.34
q35	Macronutrients	0.92	0.33
q36 *	**Macronutrients**	**0.85**	**0.26**
q37 *	**Macronutrients**	**0.54**	**0.001**
q38	Macronutrients	0.3	0.6
q39 *	**Specific Knowledge**	**0.89**	**0.15**
q40 *	**Specific Knowledge**	**0.79**	**0.27**
q41 *	**Specific Knowledge**	**0.55**	**0.2**
q42 *	**Specific Knowledge**	**0.57**	**0.19**
q43	Specific Knowledge	0.99	0.17
q44 *	**Specific Knowledge**	**0.9**	**0.11**
q45	Specific Knowledge	0.88	0.02
q46	Specific Knowledge	0.99	0.39
q47	Specific Knowledge	0.92	0.35
q48 *	**Specific Knowledge**	**0.71**	**0.23**
q49 *	**Specific Knowledge**	**0.89**	**0.31**
q50	Specific Knowledge	0.99	0.45

**Table 3:** Internal reliability calculated as Cronbach’s alpha and test–retest reliability calculated as Spearman’s correlation coefficient of the first and second survey among the same participants. The first and second surveys were separated by at least two weeks. ** *p* < 0.01.

Section	Items	Internal Reliability (Cronbach’s Alpha)	Correlation Coefficient
Total	29	0.82	0.61 **
Sub-section 1 (Energy and Weight Management)	6	0.58	0.552 **
Sub-section 2 (Macronutrients)	16	0.74	0.654 **
Sub-section 3 (Specific Knowledge)	7	0.44	0.74 **

**Table 4:** Results of the construct validity test comparing nutritional knowledge scores of focus and target groups. Results are presented as mean ± standard deviation (SD). *p*-value is the significance of the difference.

Scale	Student Focus Group	Athlete and Coach Target Group	*p*-Value
	Correct Answers (%) Mean ± SD	Correct Answers (%) Mean ± SD	
Total	77 ± 11	62 ± 18	<0.001
Energy and Weight Management	75 ± 20	59 ± 27	0.005
Macronutrients	75 ± 11	58 ± 20	<0.001
Specific Knowledge	83 ± 15	72 ± 20	0.013

## Data Availability

Data are available on request.
